# Stressful Events, Psychological Distress and Well-Being during the Second Wave of COVID-19 Pandemic in Spain: A Gender Analysis

**DOI:** 10.1007/s11482-022-10140-1

**Published:** 2022-12-31

**Authors:** M. Pilar Matud, Mª José del Pino, Juan Manuel Bethencourt, D. Estefanía Lorenzo

**Affiliations:** 1 Department of Clinical Psychology, Psychobiology and Methodology, Facultad de Psicología y Logopedia, Universidad de La Laguna, Apartado 456, 38200 La Laguna, Spain; 2 Department of Sociology, University Pablo de Olavide, Seville, Spain

**Keywords:** COVID-19, Well-Being, Psychological distress, Stress, Self-Esteem, Social support

## Abstract

The present study investigates gender differences in stressful events, psychological distress and well-being during the second wave of COVID-19 in Spain, analyzing women’s and men’s risk and resilience factors for psychological distress and for well-being. Participants were 1758 individuals from the general population, 50.8% women, aged between 18 and 79 years. Women and men did not differ in age, number of children, educational level, occupation or marital status. The participants were assessed by seven self-report questionnaires and scales. The results revealed that women experienced more psychological distress, more negative feelings, more stressful events related to the COVID-19 pandemic, more social support, and lower thriving and self-esteem than men. Multiple regression analyses showed that, in the case of women and men as well, self-esteem was the most important predictor of higher well-being and lower psychological distress and negative feelings while more COVID-19 pandemic-related stressful events were associated with higher psychological distress and lower well-being. Another important predictor of greater well-being for either gender was social support while unemployment was associated with lower well-being. In women and men, a higher educational level was associated with greater psychological distress and negative feelings; the male sample revealed that psychological distress was also connected to younger age while in women it was associated with lower instrumental social support. The results suggest that gender plays an important role in the mental health effects of the COVID-19 pandemic, with the risk being higher for women than for men.

## Introduction

Coronavirus disease 2019 (COVID-19), caused by SARS-CoV-2, was first reported in Wuhan City, China, in December 2019. This new coronavirus spread rapidly around the world, to the point that in January 2020 the World Health Organization (WHO) declared the outbreak of COVID-19 disease to be a public health emergency of international concern. With more than 118,000 cases in 114 countries, and 4,291 people dead in early March 2020, the WHO declared the COVID-19 outbreak as a pandemic on March 11, 2020 (WHO, 2020a). It has become one of the central health crises of the current generation and has affected people across all nations, continents, and socioeconomic groups (Shanafelt et al., 2020), making deep changes on all aspects of society, including physical and mental health (Holmes et al., 2020).

The rapid spread of COVID-19 and the severe nature of its symptoms led many countries to impose strict restrictions such as quarantining entire communities, closing of schools, social isolation and orders to stay at home (Shanafelt et al., 2020). The relevance of how to get people to accept and respect the norms recommended by WHO and developed by country governments to curb the spread of COVID-19 is worth considering. Recent studies have investigated the role of personality traits in facilitating or curbing the spread of COVID-19 (Espinosa & Clemente, 2021) as people with a high level of dark traits ignore the negative effects of their behaviors towards other people, show less empathy and are less inclined to accept social norms. Research shows that people with high scores on the dark triad (machiavellianism, narcissism, and psychopathy) are less compliant with COVID-19 measures of isolation and constraints (Miguel et al., 2021; Zajenkowski et al., 2020). Although not all people may have complied with the rules, the COVID-19 pandemic has significantly affected public and private life and has also entailed important social and economic implications (Haleem et al., 2020; Nicola et al., 2020). As recognized by Nicola et al. (2020), social distancing, self-isolation and travel restrictions led to a reduced workforce across all economic sectors and the loss of many jobs.

Spain was one of the European countries worst affected by COVID-19 in 2020, which resulted in approximately 80,000 excess deaths in that year, the Spanish population being 47 million (Soriano et al., 2021). The first case of coronavirus in Spain was diagnosed on January 31, 2020 and the COVID-19 pandemic spread rapidly through the country. To limit the spread of the virus, the Spanish government implemented on March 15, 2020, a national lockdown that involved a series of very restrictive measures and declared, by Royal Decree 463/2020 of March 14, 2020, a State of Alarm throughout the national territory, which was extended until June 21, 2020. Although in June 2020 these measures began to slowly relax with the implementation of the different phases of the Plan for the Transition to a "new normality", these restrictions had a profound impact on daily life, employment and the Spanish economy, since the most affected sectors (hotels, restaurants, transportation and leisure) have a greater relevance in Spain GDP than in other European countries. Whereas the strict lockdown and home confinement managed to abate the first wave of the pandemic, the overlooking of measures during the summer of 2020 entailed a second wave of COVID-19 from mid-September to Christmas 2020 (Soriano et al., 2021). Although a new State of Alarm was declared on October 25 to contain the spread of infections caused by SARSCoV-2 (Royal Decree 926/2020, October 25) and generalized measures such as social distancing, use of face masks, promotion of outdoor activities and restrictions on gatherings were established, the establishment of stricter measures was applied more flexibly and locally depending on the evolution of the pandemic so there was no longer a national closure of schools and universities nor businesses. An important aspect of this second wave in Spain was that the clinical severity of SARS-CoV-2 infection declined significantly as compared to the first wave (Soriano et al., 2021).

## Stress, Psychological Distress and Well-being during the COVID-19 Pandemic

Pandemics like COVID-19 can be mentally demanding due to the unpredictability of the situation, the uncertainty about the time the disease will be under control, and the seriousness of the risk (Al Mutair et al., 2021). These can put immense mental pressure on individuals and societies, being a time of crisis that generates stress throughout the population (Aknin et al., 2022; Chair et al., 2021; WHO, 2020b). The unexpected outbreak of COVID-19 and the public health actions imposed on the population to curb the spread of the pandemic (namely quarantine, isolation and social distancing) brought about important changes in lifestyle. These sudden and unexpected changes can be overwhelming and be the cause of stress and negative emotions in people, whose health is compromised physically and mentally too, whether they have been infected or not (Bhattacharjee & Ghosh, 2022).

While the COVID-19 pandemic has impacted on most people in some way, not everyone has experienced pandemic-related stressors (namely work, family, financial or relationship problems) to the same extent (Ciciurkaite et al., 2022). Research on stress during the COVID-19 pandemic has shown that estimates of stress prevalence vary widely and that stress prevalence can be amplified or attenuated by sociodemographic characteristics such as age, gender, or occupation. A systematic review conducted from the beginning of the COVID-19 pandemic to 17 May 2020 found that the prevalence of overall stress ranged from 8.1% to over 81.9% (Xiong et al., 2020) and among the predictors of higher stress were being female, being young, being a student and being unemployed. In a study conducted between March 17 and May 1, 2020, with respondents from 48 countries, elevated stress levels were identified with significantly higher scores among women, young people, students, and those who expressed concern about getting infected and considered themselves to be at high risk (Gamonal-Limcaoco, 2022). Changes in routine and lifestyle resulting from the pandemic have been acknowledged as particularly stressful among students since they are directly affected by school and university closures, isolation measures, leisure center closures, and social distancing, or any other measures affecting major areas of functioning (peer interaction, studies, family life and leisure) in adolescents and young adults (Ata et al., 2021; Gewalt et al., 2022; Hagedorn et al., 2022).

Although increased stress during a pandemic is to be expected, the long-term implications of this increased stress is cause of concern (Cooke et al., 2020). The psychophysiological processes involved in stress consist of bidirectional patterns of communication between the brain and the autonomic, cardiovascular, and immune systems through neural and endocrine mechanisms that support cognition, experience, and behavior. These psychophysiological processes may promote short-term adaptation (allostasis); yet they can also lead to long-term dysregulation of allostasis by promoting maladaptive wear and tear on the body and brain (allostatic load) in chronically stressful conditions (McEwen, 2016; McEwen & Gianaros, 2010), to the point of compromising stress resiliency and health. The multiple psychological stressors ensuing from health, social, economic and individual consequences related to COVID-19 may cause psychological distress, therefore, an increase in psychological distress and negative consequences for the mental health of the population can be assumed (Petzold et al., 2020; United Nations, 2020).

Psychological distress is widely used as an indicator of the mental health of the population and is largely defined as a state of emotional suffering characterized by symptoms of depression and anxiety. These symptoms may partner with somatic symptoms such as lack of energy and insomnia, which are likely to vary across cultures (Drapeau et al., 2012). Although many people experience low to moderate levels of distress in daily life, high levels can cause mental illness (Aknin et al., 2022).

Studies from several countries around the world during the first months of the COVID-19 pandemic have reported high rates of psychological distress (Kola et al., 2021; Petzold et al., 2020; Qiu et al., 2020; Xiong et al., 2020), although results indicated substantial heterogeneity among studies, even in studies that use the same instrument to assess distress (the 12-item General Health Questionnaire), the same scoring system, and the same threshold (≥ 4). For example, Daly and Robinson (2021a) found in April, 2020 in a probability sample survey of the UK that the prevalence of clinically significant psychological distress was 29.5% while in a study conducted in Belgium in March 2020 the prevalence was 65.49%, with the highest rates in women (78.09%) and in students (66.82%) (Rens et al., 2021). Other cross-sectional studies have also revealed higher rates of psychological distress in women than in men (e.g., Qiu et al., 2020; Xiong et al., 2020).

Population-based longitudinal studies conducted in the USA and the UK have shown that there was an increase in psychological distress during the first months of the COVID-19 pandemic followed by a decline to pre-pandemic levels a few months later (Daly & Robinson, 2021a, b; Riehm et al, 2021). It has been suggested that the reason underlying this rapid recovery was people’s ability to adapt to circumstances (Fancourt et al., 2021), in addition to other potential influences such as decreased perceived health risks associated with the virus and decreased financial worries as the severity of the pandemic eased; or that the decline in enforced isolation following the easing of lockdown restrictions may have alleviated psychological distress (Daly & Robinson, 2022; Robinson & Daly, 2021). But a longitudinal study with a nationally representative sample of UK adults laid bare that the prevalence of clinically significant psychological distress increased during the second wave of the COVID-19 pandemic to reach levels comparable to those observed immediately following the first wave of COVID-19 pandemic. In April 2020, following the first wave of the pandemic, the prevalence of clinically significant psychological distress in UK increased from pre-pandemic levels of 20.8% in 2019 to 29.5% in April 2020, and then declined significantly between July and September 2020 to approximately pre-pandemic levels (Daly & Robinson, 2021a, 2022). However, during late 2020/early 2021 there was a second wave of COVID-19 infections that in the UK resulted in a national social lockdown and the prevalence of clinically significant psychological distress levels increased from 21.3% in September 2020 to 27.1% in January 2021 (Daly & Robinson, 2022). Although the rise in psychological distress in the first months after the pandemic occurred in all demographic groups, the increase scored higher in women than in men (Daly & Robinson, 2021a; Etheridge & Spantig, 2022; Pierce et al., 2020). And, according to the Etheridge & Spantig, the decline in mental health in UK after the onset of the COVID-19 pandemic was more than twice as large for women than for men, and the growth of the gender gap in mental health was such large in the second wave of COVID-19.

The analysis of within-individual changes in longitudinal studies showed the existence of subgroups with different mental health responses in the first months of the COVID-19 pandemic and, although in the case of some people their mental health deteriorated, in others it did not change and in others it improved (Ahrens et al., 2021; Etheridge & Spantig, 2022; Shevlin et al., 2021). Using a longitudinal, probability sample survey Pierce et al. (2021) found that between April and October 2020, the mental health of most UK adults remained resilient although one in nine individuals had consistently poor mental health or deteriorating. In a longitudinal study carried out in Argentina in which people were assessed every 3–5 months between April 2020 and August 2021, it was reported that, although the means in psychological distress tended to increase over time, most of the individuals had consistently good mental health while remaining resilient, but a vulnerable group was detected, whose health deteriorated over time (Fernández et al., 2022).

The COVID-19 outbreak is not only a greater risk for citizens to develop psychopathology or psychological distress, but it is also a threat to their well-being (Choi et al., 2021; Khan et al., 2021). According to WHO (2022), mental health is a state of mental well-being that enables people to cope well with the many forms of life stress, realize their abilities, learn well and work well, and to contribute to their community. In fact, “good mental health is critical to each country’s response to, and recovery from, COVID-19” (United Nations, 2020, p. 5).

Traditional mental health models have focused on psychopathology, psychological problems and distress, so mental health has been defined as the absence of psychopathologies (Westerhof & Keyes, 2010). But in recent decades, research has shown that psychopathology and subjective well-being are not simply opposite poles of a single continuum (Greenspoon & Saklofske, 2001), thus acknowledging that mental illness and positive mental health are two correlated but distinct domains of mental health, each of them having shared and unique predictors (Iasiello et al., 2020; Keyes, 2003). The dual-model of mental health (cf. Greenspoon & Saklofske, 2001) suggests that psychopathology and well-being are important and distinct aspects of mental health and emphasizes the importance of incorporating positive indicators of well-being (i.e. positive affect and life satisfaction) along with traditional negative factors (i.e. mental health symptoms) for the comprehensive assessment of mental health (Antaramian et al., 2010; Greenspoon & Saklofske, 2001; Westerhof & Keyes, 2010).

There are various approaches to consider the definition and measuring of well-being (Cooke et al, 2016; Diener et al., 2017; Lindert et al., 2015; Linton et al., 2016). According to Campion (2019), mental well-being comprises several components, including affective well-being, that refers “to present experienced state of wellbeing not solely focused on pleasure”, evaluative well-being, defined “as a global, long-term assessment of wellbeing reflective of quality of life, not at a given point of time but instead over the life course” and engagement (p. 20). Huppert (2009) posits that psychological well-being is “about lives going well. It is the combination of feeling good and functioning effectively” (p. 37). Huppert considers that feeling good incorporates not only the positive emotions of happiness and contentment, but also emotions such as interest, confidence, engagement and affection; functioning effectively involves developing one's potential, having control over one's life, having a sense of purpose, and experiencing positive relationships. Beyond being a valuable outcome in itself, research has supported associations between mental well-being and a range of health benefits including increased survival (Campion et al., 2019; Diener et al., 2017; Lyubomirsky et al., 2005; Steptoe et al., 2015).

Although most studies discussing the effect of the COVID-19 pandemic on the mental health of the population have focused on the analysis of the presence of mental health problems, well-being has been assessed in several studies. The results indicated highly heterogeneity among studies, as results vary depending on the component of well-being assessed, the measure used and the country in question (Aknin et al., 2022; Anglim & Horwood, 2021; Helliwell et al., 2021; VanderWeele et al., 2021). The Gallup World Poll, which collects data from 95 countries, makes clear that, although there were considerable variations among countries, emotions changed more than life satisfaction during the first year of COVID-19. Comparing responses from 2020 to average responses from 2017 to 2019, there was no overall change in positive affect but for a 10% increase in the number of people who reported having been sad or worried the previous days (Helliwell et al., 2021). There were no significant changes in life evaluations either in 2020 or 2021 as compared to 2017–2029 (Helliwell et al., 2022). Even though it has been suggested that some components of well-being such as life satisfaction have shown resilience against COVID-19 (Aknin et al., 2022; Helliwell et al., 2022), some studies have found that life satisfaction has come down as compared with pre-pandemic times (Aknin et al., 2022; Anglim & Horwood, 2021; Fujiwara et al., 2020; VanderWeele et al., 2021; Wanberg et al., 2020), and that such decrease is reported greater in women (Milicev et al., 2022).

A decline in other components of well-being after the COVID-19 pandemic has also been identified in some studies, although the impact of the COVID-19 pandemic appears to have been different for different groups of people. For example, in a survey conducted in April and May 2020 involving 1,756 people from the general population of Pakistan in which well-being was assessed using the WHO-5 Well-being Index, the prevalence of poor well-being was 41.2% (Khan et al., 2021); moreover, poor well-being was significantly associated with sociodemographic characteristics such as being a woman, being a student, and being unemployed (Khan et al., 2021). A study conducted in Denmark using the same scale found that the well-being of the Danish population in general was negatively affected by the COVID-19 pandemic, and more so in women than in men (Sønderskov et al., 2020). And a large multi-wave survey across the European Union observed that mental well-being has dropped since summer 2020, and although the decline in well-being was across all age groups it was especially prominent among young people and those who had lost their job (Ahrendt et al., 2021).

## The Present Study

The present study investigated gender differences in stressful events related to the COVID-19 pandemic, psychological distress and well-being during the second wave of the COVID-19 pandemic in Spain. A second aim was to analyze the relevance of sociodemographic characteristics, the number of stressful events related to the COVID-19 pandemic, self-esteem and the perceived social support as risk and resilience factors of women’s and men’s psychological distress and well-being. As we count with various definitions and measures of well-being (e.g. Lindert et al., 2015) and there is considerable disagreement on the way to accurately understand and measure well-being (Cooke et al., 2016), we decided to draw on Lindert et al.'s (2015) recommendation of concurrent evaluation of at least three self-reported subjective well-being measurement scales and use three scales to measure well-being by: one assessing the cognitive component of subjective well-being, the Satisfaction with Life Scale (SWLS, Diener et al., 1985), another that evaluates the affective component, the Scale of Positive and Negative Experience (SPANE, Diener et al., 2010), and the Brief Inventory of Thriving (BIT, Su et al., 2014). According Su et al. (2014) “the term Thriving denotes the state of positive functioning at its fullest range—mentally, physically, and socially” (p. 256).

Although many studies have been conducted around the world on distress during the COVID-19 pandemic, few studies have analyzed the effect of the second wave of the pandemic in mental health (Zhang et al., 2021) and in well-being. Most cross-sectional studies have been carried out at the start of the pandemic, usually in the early stages of lockdown. In addition, as cited, there was considerable heterogeneity across studies in the distress levels found, even when using the same instrument to measure psychological distress (e.g., Pierce et al., 2020; Rens et al., 2021). There was also heterogeneity in the results of longitudinal studies, and although some suggested that the increase in psychological distress during the first few months after the pandemic was transient and that a few months later people recovered pre-pandemic levels of distress (Daly & Robinson, 2021a, b; Riehm et al, 2021), which may indicate that people had adapted to the circumstances (Fancourt et al, 2021), a longitudinal study in the UK found that during the second wave of COVID-19 the level of distress increased to levels similar to the first wave (Daly & Robinson, 2021a, 2022). Although it has been suggested that the effects of COVID-19 on mental health may be short-lived, with mental health deterioration recovering when social restrictions were eased, there is evidence that the mental health of the population has been persistently worse after the COVID-19 pandemic than before, although the deterioration in mental health varies according to sociodemographic characteristics (Patel et al., 2022).

An important limitation of previous studies is that most have not had a gender-sensitive approach and have not presented gender-disaggregated data (Flor et al., 2022). There is evidence that the health, social and economic crisis generated by the COVID-19 pandemic is having gendered impacts (Chang, 2020; Flor et al., 2022; Morgan et al., 2022; Shreeves, 2021); in addition, it has been argued that, without a gender-sensitive approach, the pandemic could have far-reaching consequences, including a real risk of exacerbating gender inequalities and reversing the progress made (Shreeves, 2021). Therefore, the present study will be conducted following a gender perspective in which, in addition to presenting and comparing the scores of women and men on the study variables, the analysis of risk and protective factors for psychological distress and well-being will be tested separately in the women's and men's groups, thus presenting data disaggregated by gender. Given that there is evidence that sociodemographic characteristics are relevant in stress, psychological distress and well-being experienced during the COVID-19 pandemic, the study will ensure that women and men present similar sociodemographic characteristics.

Based on previous findings, we hypothesized that women will have higher stress (Gamonal-Limcaoco, 2022; Kowal et al., 2020; Xiong et al., 2020), greater psychological distress (Etheridge & Spantig, 2022; Milicev et al., 2022; Pierce et al., 2020; Qiu et al., 2020; Rens et al., 2021; Xiong et al., 2020) and lower well-being (Khan et al., 2021; Sønderskov et al., 2020) than men. Given that there is evidence that stress is associated with distress and is a threat to well-being (Petzold et al., 2020; United Nations, 2020), the second hypothesis of the study posits that women and men who have experienced more stressful events related to the COVID-19 pandemic will score higher in psychological distress and lower in well-being. Finally, since there is evidence that social support (Li et al., 2020; Matud et al., 2022; Petzold et al., 2020; Yalçın et al., 2022) and high self-esteem (Matud et al., 2022; Rossi et al., 2020a, b; Zhao et al., 2022) are protective factors for mental health during the COVID pandemic, we hypothesized that women and men with higher self-esteem and higher social support will present lower psychological distress and higher well-being (third hypothesis).

## Method

### Participants and Procedure

This study used a cross-sectional design and was conducted during the second wave of COVID-19 in Spain, from late October to early December 2020. Participants were 1758 individuals from the general population, 50.8% (*n* = 893) women and 49.2% (*n* = 865) men aged between 18 y 79 years (*M*_age_ = 32.79, *SD* = 15.65). Table 1 displays the main sociodemographic characteristics of the participants. There was diversity in their educational levels, although people with a level of education lower than secondary school are in the minority. Furthermore, almost half of the sample (44.6%) were employed and 40.5% were students. And virtually half of the sample (51.6%) were single and did not live with a partner. As can be seen in Table 1, women and men did not differ significantly in age, number of children, educational level, occupation and marital status.

**Table 1:** Demographic characteristics of the male and female groups

	Male		Female		*χ*^*2*^	*p*
Variables	*n*	*%*	*n*	*%*		
*Education:*					1.07	.90
Without studies	7	0.8	7	0.8		
Primary school	55	6.4	62	6.9		
Secondary school	110	12.7	101	11.3		
High-school degree	356	41.2	366	41.0		
University degree	337	39.0	357	40.0		
*Occupation:*					0.36	.94
Student	356	41.2	356	39.9		
Unemployed	78	9.0	81	9.1		
Retired	51	5.9	52	5.8		
Employed	380	43.9	404	45.2		
*Marital status:*					0.37	.83
Never married	449	51.9	458	51.3		
Married/partnered	363	42.0	374	41.9		
Separated/divorced/widowed	53	6.1	61	6.8		
	*M*	*SD*	*M*	*SD*	*t*	*p*
Age	32.77	15.78	32.81	15.53	-0.06	.95
Number of children	0.56	0.97	0.60	0.92	-0.75	.45

All participants were volunteers and did not receive remuneration for their participation. A convenience and snowballing recruitment strategy was adopted for sampling. Access to the participants was through the social network of researchers and university students of psychology and sociology from three Spanish universities who received course credits for the task. All data were collected through an online survey using a Google Form. Participants were sent an electronic link to complete the online survey from their personal computers or on their mobile phones using the WhatsApp application at their convenience. The female sample in this study was randomly selected from a larger sample on the psychological impact of the second wave of the COVID-19 pandemic in Spain, following the criterion that women and men did not present statistically significant differences in age, number of children, level of education, occupation, and marital status. Given that the number of women who had completed and returned the questionnaires in that time period was much greater than that of men, the male sample was drawn first and, once their demographic characteristics were known, a similar number of women who did not differ significantly from the male sample were selected. The procedures of this study complied with the ethical standards of the 1964 Helsinki declaration and its later amendments and also met the ethical criteria of the American Psychological Association. All participants voluntarily gave their informed consent to participate in the study after being informed about the aims of the study, confidentiality, and right to withdraw at any time. The research was approved by the Ethics Committee for Human Research (CEIH) of the Pablo de Olavide University of Seville (code 21/8–6).

### Instruments

*Sociodemographic characteristics*. Information was collected on gender (male, female or other), age, marital status, number of children, educational level and occupation.

*Scale of Positive and Negative Experience* (SPANE, Diener et al., 2010) is a 12-item scale designed to assess “the full range of possible desirable and undesirable experiences” (Diener et al., 2010, p. 145). Six items assess positive feelings (SPANE-P, items: positive, good, pleasant, happy, joyful, contented) and six items assess negative feelings (SPANE-N, items: negative, bad, unpleasant, sad, afraid, angry). Items are scored on a scale ranging from 1 (very rarely or never) to 5 (very often or always) and the positive and the negative scales are independently scored. SPANE has been validated in many countries and has shown good psychometric properties and a correlated two-factor structure (Jovanović et al., 2020; Li et al., 2013; Silva & Caetano, 2013). In the present study, the Cronbach’s α reliability test for SPANE-P had a value of 0.89 and 0.84 for SPANE-N.

*Satisfaction with Life Scale* (SWLS; Diener et al., 1985). This scale was developed to assess global life satisfaction, which is considered to be the cognitive component of subjective well-being. The scale consists of five questions which are rated on a scale from 1 (strongly disagree) to 7 (strongly agree). Example items are “In most ways my life is close to my ideal”, “I am satisfied with my life”. The SWLS is a widely used instrument that has been validated in many countries and has demonstrated adequate psychometric properties (see review in Pavot & Diener, 2009). The Cronbach’s α in the sample of the present work was 0.85.

The *Brief Inventory of Thriving* (BIT, Su et al., 2014) is a 10-item inventory designed to swiftly assess positive psychological health “measuring the core psychological well-being dimension” (Su et al., 2014, p. 256). Example items are “My life has a clear sense of purpose”, “I am optimistic about my future”, “I am achieving most of my goals”. Items are rated on a scale from 1 (strongly disagree) to 5 (strongly agree). In validation studies the BIT has demonstrated sound psychometric properties and convergent validity with existing measures of psychological well-being (Arslan, 2021; Duan et al., 2016; Sorgente et al., 2021; Su et al., 2014; Wiese et al., 2018). For the present sample, the Cronbach’s α was 0.89.

*The General Health Questionnaire*-12 item version (GHQ-12, Goldberg et al., 1996) is a 12-item screening instrument and a popular measure of psychological distress (Gnambs et al., 2018). Participants rate how often they have experienced each symptom in the past few weeks. The questionnaire contains items such as “Felt you couldn't overcome your difficulties”, "Felt constantly under strain". The GHQ-12 has been applied to various populations in different countries and has demonstrated strong psychometric properties (Gelaye et al., 2015; Liang et al., 2016). In this study, item scores were coded according to the Likert method (0–1-2–3), and the standard GHQ scoring method (0–0-1–1). The Likert score was used in all analyses, except for discriminating distressed from non-distressed cases, where the standard GHQ score was used. According to Lundin et al. (2017) the best threshold for discriminating distressed from non-distressed cases for the GHQ Index was ≥ 4 (sensitivity = 81.7 and specificity = 85.4). In this study, the Cronbach's α of 12 items, scored according to the Likert method, gave a value of 0.88, whereas it was 0.86 after the standard GHQ method.

*The Rosenberg Self-Esteem Scale* (RSES, Rosenberg, 1965) is a 10-item scale that assesses global self-esteem. The RSES has been validated in many countries and is the most widely used instrument to assess self-esteem (Monteiro et al., 2022). The scale contains items such as “I am able to do things as well as most other people” and “I take a positive attitude toward myself”. All items were answered on a 4-point scale ranging from 0 (strongly agree) to 3 (strongly disagree). In this study, the Cronbach’s α was 0.84.

*The Social Support Scale* (Matud, 1998) is a 12-item self-report measure that assesses perceived availability of social support. Items are structured into two factors: Emotional support, consisting of seven items (e.g., “Someone who comforts you when you are upset”) and instrumental support (e.g., “Someone who lends you money when you have economic problems”). Items are rated on a 4-point scale from 0 (never) to 3 (always). For the current sample, the Cronbach’s α for the emotional support was 0.90 and for the instrumental support was 0.85.

*Stressful events during the COVID-19 pandemic.* To find out if people had experienced stressful events, in addition to the daily problems and inconveniences of the Coronavirus pandemic, they were asked if the following events and/or losses had happened to them since the pandemic began: 1) job loss, 2) economic problems, 3) major arguments with partner, 4) major arguments with family, 5) illness of relatives and/or loved ones, 6) death of one or more relatives and/or loved ones, 7) personal illness. Moreover, they were asked to describe any other event(s) and/or losses they experienced.

### Data Analysis

Statistical analyses were conducted using IBM SPSS Statistics for Windows, version 21.0 (IBM Corp., Armonk, N.Y., USA). The comparison between women and men in quantitative variables was calculated using Student's t-test and comparisons in education, marital status, occupation and whether or not they were GHQ distressed cases using Pearson's Chi-square test. The effect size of the quantitative differences was calculated using Cohen's *d*. Correlation analyses based on Pearson’s correlation coefficients were performed to explore relationships between measures of well-being and psychological distress, and a Principal Components analysis was carried out to reduce the number of variables that assess well-being and psychological distress to a smaller number of components.

By analyzing separately the samples of women and men, hierarchical multiple regression analyses were performed to determine the relevance of sociodemographic characteristics, the number of stressful events related to the COVID-19 pandemic, the self-esteem and the social support in well-being and in psychological distress. The criterion in the first regression analysis was the total score on psychological well-being and in the second analysis the score on distress and negative feelings. In each regression analysis, age and number of children were included in the first step as quantitative variables, education as an ordinal variable with five levels (without studies, primary school, secondary school, high school and university degree), and marital status and the profession as dummy variables. In marital status, not having a partner was considered as a reference category, coding 0 for never married and for separated, divorced and widowed people, and married or partnered people was coded with 1. In occupation, two dummy variables were made: 1) unemployed, coding unemployment with 1 and with 0 for the rest of the occupation categories (students, retired, employed) that were considered as the reference variable; 2) student, coding students with 1 and with 0 the rest of labor categories (unemployed, retired, employed). A significance level of 95% was used as criteria in comparison, correlation and regression analyses.

## Results

Table 2 displays the means, standard deviations and comparisons between women and men in the study variables. As can be seen, although the effect size was small or trivial, there were statistically significant differences in all the variables, except for positive feelings, life satisfaction and well-being. Women scored higher than men in psychological distress, negative feelings, number of stressful events related to the COVID-19 pandemic and instrumental and emotional support, while men scored higher than women in thriving and self-esteem.

**Table 2:** Means (*M*), standard deviations (*SD*) and comparisons between the male and the female samples for the study variables

	Male (*n* = 865)		Female (*n* = 893)		*t*	*d*
Variables	*M*	*SD*	*M*	*SD*		
Psychological distress	14.05	6.53	16.10	6.77	−6.47***	0.31
Negative feelings	15.48	4.41	17.14	4.13	−8.16***	0.39
Positive feelings	20.95	4.12	20.72	4.03	1.16	–
Life satisfaction	23.62	6.40	23.65	6.34	−0.09	–
Thriving	37.52	6.43	36.74	6.52	2.52*	0.12
Self-esteem	20.27	4.71	19.39	4.92	3.82***	0.18
Emotional support	16.05	4.65	16.85	4.38	−3.72***	0.18
Instrumental support	9.67	3.82	10.71	3.73	−5.80***	0.28
Number of stressful events	1.48	1.37	1.66	1.42	−2.70**	0.13
Well-being	82.09	14.46	81.11	14.72	1.40	–
Psychological distress and negative feelings	29.52	9.89	33.24	9.93	−7.86***	0.37

^*^p < .05, **p < .01, ***p < .001

More than half of the sample (56.5%) had scores that exceeded the cut-off point to be classified as a distressed case, a percentage that was higher in women (63.4%) than in men (49.5%), χ^2^(1, *N* = 1758) = 34.56, *p* < 0.001. Statistically significant differences (*p* < 0.001) were also found in the percentage of people who could be classified as a distressed case as for occupation, with the highest percentage being students (65.0%) and unemployed (63.5%) and the lowest in retired people (39.8%). 71.3% of female students and 58.7% of male students experienced psychological distress, differences that were statistically significant χ^2^(1, *N* = 712) = 12.51, *p* < 0.001. Among those who were unemployed, 65.4% of the women and 61.5% of the men underwent psychological distress, differences that were not statistically significant χ^2^(1, *N* = 159) = 0.26, *p* = 0.61. Among employed people, 57.7% of women and 41.1% of men presented psychological distress, differences that were statistically significant χ^2^(1, *N* = 784) = 21.64, *p* < 0.001. In the case of retired people, 50.0% of women and 29.4% of men experienced psychological distress, differences that were statistically significant χ^2^(1, *N* = 103) = 4.56, *p* = 0.033.

Analysis of stressful events experienced since the pandemic began showed that 75.3% of women and 70.8% of men reported experiencing one or more COVID-19 pandemic-related stressful events. The most frequently cited was illness of relatives and/or loved ones, cited by 38.7% of women and 32.8% of men, differences that were statistically significant χ^2^(1, *N* = 1758) = 6.68, *p* = 0.01. This was followed in frequency by economic problems, cited by 28.2% of women and 24.7% of men, differences that were not statistically significant χ^2^(1, *N* = 1758) = 2.71, *p* = 0.10. The next most cited event was major arguments with family, cited by 26.4% of women and 23.1% of men, differences that were not statistically significant χ^2^(1, *N* = 1758) = 2.58, *p* = 0.11. Death of one or more relatives and/or loved ones was cited by 26.9% of women and 22.5% of men, differences that were statistically significant χ^2^(1, *N* = 1758) = 4.43, *p* = 0.03. Major arguments with partner was cited by 15.5% of women and 14.5% of men, differences that were not statistically significant χ^2^(1, *N* = 1758) = 0.35, *p* = 0.56. Job loss was cited by 11.3% of women and 12.8% of men, differences that were not statistically significant χ^2^(1, *N* = 1758) = 0.96, *p* = 0.33. The least frequently cited event was personal illness, by 11.0% of women and 10.9% of men, differences that were not statistically significant χ^2^(1, *N* = 1758) = 0.01, *p* = 0.94. In addition, 8.0% of women and 6.6% of men reported experiencing during the COVID-19 pandemic an event other than those cited, differences that were not statistically significant χ^2^(1, *N* = 1758) = 1.21, *p* = 0.27.

The correlation analysis between measures of well-being and psychological distress revealed statistically significant correlations (*p* < 0.001). As can be seen in Table 3, in women and men positive feelings (SPANE-P) was positively correlated to thriving (BIT) and to life satisfaction (SWLS), and BIT also was positively related to SWLS. Negative feelings (SPANE-N) was positively related to psychological distress (GHQ-12), and negatively related to SPANE-P, to SWLS and to BIT; and psychological distress was negatively related to SPANE-P, SWLS and BIT. To reduce the number of variables to a smaller number of components, a Principal Components analysis was conducted. The Kaiser–Meyer–Olkin index of sampling adequacy was 0.96, and the Bartlett sphericity test was *p* < 0.001. The Scree test (Cattell, 1978) asymptoted at two factors, so the analysis was performed requesting two components and varimax rotation. The first component explained 22.81% of the rotated matrix variance and the 10 items of the BIT, the 5 items of the SWLS and the 6 items of the SPANE-P loaded above 0.40. The items that loaded the highest were item 5 of the BIT (“What I do in life is valuable and worthwhile”) and item 3 of the SWLS (“I am satisfied with my life”) with component loading of 0.71 and those with the lowest loading were item 9 of the BIT (“There are people who appreciate me as a person”) and item 5 of the SWLS (“If I could live my life over, I would change almost nothing”), with a loading component of 0.54 and 0.52 respectively. The second component explained 19.65% of the rotated variance and included the 12 items of the GHQ-12 and the 6 items of the SPANE-N. The items with the highest loading (0.72) were item 5 (“Felt constantly under strain”) and item 9 (“Been feeling unhappy and depressed”) of the GHQ-12 and those with the lowest weight were item 11 of the SPANE-N (angry) and item 3 of the GHQ-12 (“Felt that I am playing a much less useful part”) whose loading were 0.47 and 0.42 respectively. The internal consistency analysis (Cronbach's alpha) of the 21 items that make up the first component, which we called well-being, was 0.93 and that of the 18 items of the second component, which we called psychological distress and negative feelings, was 0.91. Both components correlated -0.62 (*p* < 0.001).

**Table 3:** Intercorrelations between psychological distress and well-being measures disaggregated by gender

	1	2	3	4
Male sample (*n* = 865)				
1. Psychological distress	–			
2. Negative feelings	.64***	–		
3. Positive feelings	−.47***	−.50***	–	
4. Life satisfaction	−.50***	−.46***	.52***	–
5. Thriving	−.58***	−.51***	.66***	.69***
Female sample (*n* = 893)				
1. Psychological distress	–			
2. Negative feelings	.66***	–		
3. Positive feelings	−.50***	−.47***	–	
4. Life satisfaction	−.44***	−.38***	.46***	–
5. Thriving	−.54***	−.45***	.60***	.66***

^***^p < .001

Table 4 displays the main results of the hierarchical multiple regression predicting well-being scores for the male group, and Table 5 displays the results for the female group. In both groups *R* was significantly different from zero at the end of each step. The sociodemographic variables entered into step 1 explained 6% of the variance in well-being in the male group and 1% in the female group. In either gender, being unemployed was statistically significant, with lower well-being in unemployed women and men with respect to the rest of the occupations. In addition, in the male sample, student occupation and marital status were also statistically significant, with lower well-being in male students and higher well-being in married/partnered men. The addition of stressful events in step 2 resulted in a significant increase in *R*^2^ in both groups, with higher well-being among those who had experienced fewer stressful events during the COVID-19 pandemic. The addition of self-esteem, emotional and instrumental support in the regression equation (Model 3) resulted in a significant increase in *R*^2^. The adjusted *R*^2^ value of 0.51 in male group and 0.54 in the female group indicated that more than half of the variability in well-being was predicted. Beta values ​​in Model 3, with all the variables in the equation, proved that the most important variable in the prediction of well-being of women and men was higher self-esteem, followed by higher emotional support. In both groups fewer stressful events during the COVID-19 pandemic, not being unemployed and higher instrumental support were also significant predictors of well-being. In addition, in the female sample, to be a student was a significant predictor of greater well-being.

**Table 4:** Summary of the hierarchical multiple regression with well-being as the dependent variable in the male sample

	Model 1			Model 2			Model 3		
Variables	*B*	*SE B*	β	*B*	*SE B*	*β*	*B*	*SE B*	β
Age	−0.11	0.06	−.12	−0.12	0.06	−.13*	−0.02	0.04	−.02
Number of children	−0.08	0.78	−.01	0.18	0.76	.01	0.13	0.57	.01
Educational level	−0.08	0.54	−.01	−0.14	0.53	−.01	−0.05	0.39	−.00
Unemployed	−12.12	1.77	−.24***	−9.10	1.77	−.18***	−5.34	1.32	−.11***
Student	−3.41	1.41	−.12*	−3.36	1.37	−.11*	0.55	1.03	.02
Married/partnered	3.47	1.10	.12**	2.84	1.08	.10**	0.49	0.81	.02
Number of stressful events				−2.57	0.35	−.24***	−1.21	0.27	−.12***
Self-esteem							1.43	0.08	.46***
Emotional support							0.80	0.11	.26***
Instrumental support							0.31	0.14	.08*
*R*^*2*^		.07			.12			.52	
*R*^*2*^ Change		.07***			.06***			.40***	

B, unstandardized coefficient; *SE B*, standard error of B; β, standardized coefficient; R^2^, percentage of variance explained

^*^p < .05, ** *p* < .01, ***p < .001

**Table 5:** Summary of the hierarchical multiple regression with well-being as the dependent variable in the female sample

	Model 1			Model 2			Model 3		
Variables	*B*	*SE B*	β	*B*	*SE B*	*β*	*B*	*SE B*	β
Age	0.02	0.05	.02	−0.01	0.05	−.01	−0.02	0.04	−.02
Number of children	0.22	0.83	.01	0.21	0.80	.01	−0.13	0.57	−.01
Educational level	0.08	0.59	.01	0.18	0.57	.01	0.44	0.40	.03
Unemployed	−7.12	1.78	−.14***	−5.00	1.73	−.10**	−3.86	1.23	−.08***
Student	−0.44	1.44	−.02	−0.80	1.38	−.03	3.55	0.99	.12***
Married/partnered	−0.47	1.18	−.02	−0.52	1.14	−.02	−0.75	0.81	−.00
Number of stressful events				−2.89	0.34	−.28***	−1.16	0.25	−.11***
Self-esteem							1.77	0.08	.59***
Emotional support							0.55	0.13	.16***
Instrumental support							0.37	0.15	.09*
*R*^*2*^		.02			.09			.55	
*R*^*2*^ Change		.02*			.08***			.45***	

B, unstandardized coefficient; *SE B*, standard error of B; β, standardized coefficient; R^2^, percentage of variance explained

^*^p < .05, ** *p* < .01, ***p < .001

Table 6 displays the results of the hierarchical multiple regression predicting the psychological distress and negative feelings scores in the male sample and Table 7 in the female sample. In both groups *R* was significantly different from 0 at the end of each step. The sociodemographic variables entered into step 1 explained 7% of the variance in psychological distress and negative feelings in the male group and 4% in the female group. In the male sample all sociodemographic variables were statistically significant except marital status and number of children, thus showing that men with higher psychological distress and negative feelings were younger, had higher educational level, and were students or unemployed. In the female sample, only two of the variables were statistically significant: educational level and unemployed; unemployed women and those with higher educational levels presented higher psychological distress and negative feelings. In either group, the addition of number of stressful events in step 2 resulted in a significant increase in *R*^2^, with greater psychological distress and negative feelings in women and men who had experienced a higher number of stressful events related to the COVID-19 pandemic. Adding self-esteem and emotional and instrumental support to the equation (Model 3) resulted in a significant increase in *R*^2^. The adjusted *R*^2^ value of 0.42 in men and 0.38 in women indicated that more than a third of the variability in psychological distress and negative feelings was predicted. Beta values ​​in Model 3, with all variables in the equation, proved that the most important variable in the psychological distress and negative feelings of both women and men was lower self-esteem, followed by a higher number of stressful events related to the COVID-19 pandemic. Another significant predictor variable in both samples was the educational level, with higher psychological distress and negative feelings in women and men who had a higher educational level. Age was also a significant variable in the male sample, and younger men experienced greater psychological distress and negative feelings, while in the female sample instrumental support was statistically significant, as women with less instrumental support showed greater psychological distress and negative feelings.

**Table 6:** Summary of the hierarchical multiple regression with psychological distress and negative feelings as the dependent variable in the male sample

	Model 1			Model 2			Model 3		
Variables	*B*	*SE B*	β	*B*	*SE B*	*β*	*B*	*SE B*	β
Age	−0.08	0.04	−.12*	−0.07	0.04	−.10	−0.09	0.03	−.15**
Number of children	0.95	0.53	.09	0.67	0.50	.07	0.74	0.42	.07
Educational level	1.61	0.37	.15***	1.67	0.34	.15***	1.65	0.29	.15***
Unemployed	6.22	1.21	.18***	3.05	1.15	.09**	1.00	0.98	.03
Student	2.74	0.96	.14**	2.69	0.89	.13**	0.55	0.77	.02
Married/partnered	−0.81	0.75	−.04	−0.15	0.70	−.01	0.48	0.61	.02
Number of stressful events				2.69	0.23	.37***	2.10	0.20	.29***
Self-esteem							−0.96	0.06	−.46***
Emotional support							−0.16	0.09	−.08
Instrumental support							−0.01	0.10	−.01
*R*^*2*^		.08			.21			.43	
*R*^*2*^ Change		.08***			.13***			.22***	

B, unstandardized coefficient; *SE B*, standard error of B; β, standardized coefficient; R^2^, percentage of variance explained

^*^p < .05, ***p < .001

**Table 7:** Summary of the hierarchical multiple regression with psychological distress and negative feelings as the dependent variable in the female sample

	Model 1			Model 2			Model 3		
Variables	*B*	*SE B*	β	*B*	*SE B*	*β*	*B*	*SE B*	β
Age	−0.07	0.04	−.11	−0.05	0.03	−.08	−0.03	0.03	−.04
Number of children	0.03	0.55	.00	0.04	0.52	.00	0.31	0.44	.03
Educational level	1.04	0.39	.10**	0.96	0.37	.09*	0.81	0.32	.08*
Unemployed	3.26	1.19	.09**	1.58	1.13	.05	0.93	0.96	.03
Student	1.78	0.96	.09	2.08	0.90	.10*	−0.28	0.78	−.01
Married/partnered	0.43	0.79	.02	0.47	0.74	.02	−0.04	0.64	−.00
Number of stressful events				2.29	0.22	.33***	1.52	0.19	.22***
Self-esteem							−1.00	0.06	−.50***
Emotional support							0.13	0.10	.06
Instrumental support							−0.36	0.12	−.14**
*R*^*2*^		.04			.15			.39	
*R*^*2*^ Change		.04***			.11***			.24***	

B, unstandardized coefficient; *SE B*, standard error of B; β, standardized coefficient; R^2^, percentage of variance explained

^*^p < .05, ** *p* < .01, ***p < .001

## Discussion

The present study aimed to investigate gender differences in COVID-19-related stressful events, psychological distress and well-being during the second wave of the COVID-19 pandemic in Spain. In addition, it sought to analyze the relevance of sociodemographic characteristics, the number of stressful events associated with the COVID-19 pandemic, the self-esteem and the perceived social support as risk and resilience factors of women’s and men’s psychological distress and well-being. Results indicated that, although the effect size was low, and very low in the case of thriving and the number of stressful events connected to the COVID-19 pandemic, women reported more psychological distress, negative feelings, emotional and instrumental social support and had experienced a greater number of stressful events that had a bearing with the COVID-19 pandemic. In addition, they presented lower self-esteem and less thriving than men. These results confirm, although not completely, hypothesis 1, which stated that women would have greater stress, greater psychological distress and lower well-being than men. Although women scored higher than men on negative feelings and thriving, the effect size on thriving was very low, and there were no differences between women and men on two of the components of subjective well-being assessed, positive feelings and life satisfaction.

The results regarding the percentage of people who experienced psychological distress during the second wave of the COVID-19 pandemic in Spain showed higher rates in women than in men, although the percentage also varied according to occupation. In the present study, the highest percentage of people experiencing psychological distress was found in female students (71.3%) and the lowest in retired men, a group in which only 29.4% experienced psychological distress. But in all occupation groups the percentage of women experiencing mental distress was higher than that of men, although in unemployed persons the differences were not statistically significant. At least half of the women underwent psychological distress during the second wave of the COVID-19 pandemic in Spain, the percentage being lowest in retired women (50%) and highest in students (71.3%).

Taken together, these results indicate that the second wave of the COVID-19 pandemic entails a greater risk to mental health and well-being in women than in men, results that are consistent with Elsayed et al. (2022) and Etheridge and Spantig (2022) and also with findings obtained during the first wave of the COVID-19 pandemic (e.g., Fancourt et al., 2021; Gamonal-Limcaoco et al., 2022; Khan et al., 2021; Matud et al., 2022; Pierce et al, 2020; Qiu et al., 2020; Riehm et al., 2021; Sønderskov et al., 2020; Xiong et al., 2020). In addition to entailing an increase in gender differences in health risks (Connor et al., 2020), they may also increase other gender inequalities. It has been argued that gender gaps in mental symptomatology and well-being may increase inequalities among women in other areas, since, for example, “lower mental well-being can reduce productivity and thus impact current and future earnings, increasing the gender gap in pay” (Etheridge & Spantig, 2022, p. 15).

Multiple regression analyses conducted in the present study showed that women and men who had experienced a greater number of COVID-19 pandemic-related stressful events and/or changes proved to suffer from greater psychological distress and lower well-being, as hypothesized in the second hypothesis. Although, the effects on either women and men appear to be greater in predicting psychological distress and negative feelings than in predicting well-being, a greater number of events and/or stressful changes related to the COVID-19 pandemic was associated with lower well-being, results that are consistent with those of studies conducted in other countries (e.g., Ciciurkaite et al., 2022). In the present study, in the female sample, being a student was slightly more predictive of well-being than the number of stressful events, with the magnitude of Beta in the final model being 0.12 for student and -0.11 for the number of stressful events related to COVID-19. Even if there is no doubt that a greater number of stressful events related to COVID-19 are associated with lower well-being in both genders, and that some sociodemographic characteristics are also relevant in well-being, multiple regression analyses predicting women’s and men’s well-being showed that self-esteem and emotional social support were the most important predictors. In fact, although the increase in R^2^ in Model 1, where only sociodemographic variables were included, and in Model 2, where the number of stressful events was added, was statistically significant, the magnitude of the change in R^2^ from Model 2 to Model 3, where self-esteem and social support were included, was much larger, being 0.45 in women and 0.40 in men, whereas the change in R^2^ from Model 1 to Model 2 was 0.08 in women and 0.06 in men. When predicting psychological distress and negative feelings, a greater number of stressful events associated with the COVID-19 pandemic was the second most relevant variable in women and men, with low self-esteem being the most important predictor. These findings are consistent with those of other studies that have proved that greater stress was linked to greater mental symptomatology during the COVID-19 pandemic (Ahrens et al., 2021; Li et al., 2020; Rossi et al., 2020a, b).

Most of the persons in the present study sample (75.3% of women and 70.8% of men) reported experiencing one or more stressful events related to the COVID-19 pandemic. The most frequently cited was illness of relatives and/or loved ones, which was cited by approximately one-third of the sample, followed by economic problems, major arguments with family, death of one or more relatives and/or loved ones, major arguments with partner, and job loss, cited by just over 10% of the sample. Personal illness was only cited by 11.0% of the women and 10.9% of the men, which supports the assertion by other authors that the COVID-19 pandemic has generated important psychosocial stressors, although not all people have experienced the different stressors to the same extent (Ciciurkaite et al., 2022). Except for job loss, which was cited by 12.8% of men and 11.3% of women, women cited more frequently than men each of the stressful events related to COVID-19, although statistically significant differences were only found in the percentages for illness and death of relatives and/or loved ones. Also in studies conducted in other countries during the COVID-19 pandemic, stress has been found to be higher in women than in men (Gamonal-Limcaoco, 2022; Kowal et al., 2020; Rossi et al., 2020a, b; Xiong et al., 2020).

The results of the analysis of the relevance of sociodemographic characteristics on the well-being of women and men evidenced that their relevance was low, although in both genders it was found that unemployed people scored lower well-being. These results are consistent with those of other studies where unemployment has been found to be associated with lower well-being (Ahrendt et al., 2021; Khan et al., 2021). In addition, in the female sample, greater well-being was associated with being a student, which is the most important predictor of well-being after self-esteem and social support. This is an interesting result given that clinically significant psychological distress was detected in 71.3% of the female students. These results support what is proposed in the dual model of mental health, which states that mental symptomatology and well-being are not the opposite poles of the same continuum and that it is important to test both components for a comprehensive assessment of mental health (e.g., Antaramian et al., 2010; Greenspoon & Saklofske, 2001; Iasiello et al., 2020; Keyes, 2003; Westerhof & Keyes, 2010).

The analysis of the relevance of sociodemographic variables in psychological distress showed that, in both genders, a higher level of education predicted greater psychological distress, although the Beta weight of men (0.15) was almost twice that of women (0.08). Also in some studies conducted in other countries during the COVID-19 pandemic, it has been found that people with higher educational level had more mental symptomatology (Qiu et al., 2020; Wanberg et al, 2020). But the results of the relevance of the educational level on mental health during the COVID-19 pandemic are inconclusive as in several studies it has been found that people with lower educational level had worse mental health while in other studies there were no differences in mental health based on educational level (see review by Gibson et al., 2021).

The present study also evidenced that greater psychological distress was also associated with younger age in the case of men, which was not the case for women. Although other studies conducted during the COVID-19 pandemic have suggested that younger people are at higher risk (e.g., Almomani et al., 2021; Pierce et al., 2020; Rossi et al., 2020a, b), findings of this study suggest that the higher risk is limited to men. Furthermore, although most of female students (71.3%) and more than half of male students (58.7%) had clinically significant levels of psychological distress, in the final regression models predicting psychological distress it was found that, once self-esteem and social support were included in the regression equation, being a student was no longer statistically significant and its Beta weight was very close to 0 in both females and males.

Findings of this study showed that, in either gender, the most protective variable for mental health was high self-esteem, in addition to social support when it came to predicting well-being. And although in the female sample instrumental support also appeared as a protective factor against psychological distress, in the case of men neither emotional support nor instrumental support were significantly associated with less psychological distress and negative feelings. These results support, although only partially, the third hypothesis of the study, which stated that women and men with higher self-esteem and greater social support will report less psychological distress and greater well-being. Our results confirm previous finding regarding the relevance of self-esteem as a factor of resilience to the COVID-19 pandemic (Matud et al., 2022; Rossi et al., 2020a, b; Zhao et al., 2022) and, also, of social support (e.g., Ahrens et al., 2021; Gewirtz-Meydan & Lassri, 2022; Petzold et al., 2020; Yalçın et al., 2022).

## Strengths, Limitations and Further Research

Ryff and Singer (2003) argued that positive human functioning is perhaps most notable when it is evident in contexts of great life challenge and adversity, and it is then, when individuals are being tested, when much can be learned about human strengths: what they are, how they emerge, and how they are nurtured or undermined. It has also been argued that their consideration and measurement should guide policy during the pandemic and beyond (Aknin et al., 2022). Likewise, the United Nations highlights the importance of focusing on the positive aspects of mental health during the COVID-19 pandemic by stating "Good mental health is critical to each country's response to, and recovery from, COVID-19." (2020, p. 5). It has also been posited that recognizing the extent to which the pandemic has affected women and men differently is a fundamental step in understanding its primary and secondary effects on different individuals and communities and for creating effective and equitable interventions and policies (Wenham et al., 2020). The present study contributes to such knowledge by assessing different components of mental well-being and conducting a study in which gender-disaggregated results are presented in a sample consisting of women and men in which their sociodemographic characteristics have been controlled to be similar. However, the study has important limitations. Firstly, the sample is a convenience sample and therefore may not be representative of the entire Spanish population. And, although the sociodemographic characteristics are diverse, a minority of people have just basic education. Secondly, the study is cross-sectional, which does not allow us to speak of cause-effect relationships. Furthermore, all the measures were taken during the second wave of the pandemic in Spain, between late October to early December 2020, but the pandemic has been evolving for more than two years and there have been important changes, among which the vaccination of the population and the presence of successive pandemic waves stand out. Third, the study was conducted in a single country, Spain. Fourth, all the data were obtained through self-report, which can be a source of biases, including social desirability. Future studies should use random samples and be longitudinal research carried out in several countries using, in addition, a multimethod evaluation.

## Conclusions

The results of the present study showed that the second wave of the COVID-19 pandemic entails a significant risk of distress for the Spanish population, although there are important differences since the rates of clinically significant distress range from 71.3% in female students to 29.4% in male retirees. Women, as compared to men, are more at risk for mental health problems, as they have more psychological distress and negative feelings and less thriving than men. Self-esteem was shown to be an important factor in both women's and men's well-being and protection against psychological distress and negative feelings. Social support, especially emotional support, was also important in the well-being of women and men while unemployment was a risk factor. Higher educational level and younger age were risk factors for greater psychological distress in men while in women instrumental support was a protective factor for psychological distress, and higher educational level was a risk factor, although its effect was low. The results of the present study are relevant for the design of policies and programs aimed at the promotion of well-being and the prevention of psychological distress in women and men, as well as for the knowledge of the groups most at risk for mental health problems during the second wave of the COVID-19 pandemic.
